# Systemic Sentinel Lymph Node Detection Using Fluorescence Imaging After Indocyanine Green Intravenous Injection in Colorectal Cancer: Protocol for a Feasibility Study

**DOI:** 10.2196/17976

**Published:** 2020-08-14

**Authors:** Gabriel Liberale, Sophie Vankerckhove, Fikri Bouazza, Maria Gomez Galdon, Denis Larsimont, Michel Moreau, Pierre Bourgeois, Vincent Donckier

**Affiliations:** 1 Institut Jules Bordet, Belgian Comprehensive Cancer Center Université Libre de Bruxelles (ULB) BE 0257.981.101. Brussels Belgium

**Keywords:** indocyanine green, colorectal cancer, fluorescence imaging, nodal staging, sentinel lymph node detection, cancer, lymph node, prognosis, treatment

## Abstract

**Background:**

Nodal staging is a major concern in colorectal cancer as it is an important prognostic factor. Several techniques that could potentially improve patient treatment and prognosis have been developed to increase the accuracy of nodal staging. Sentinel lymph node detection has been shown to accurately reflect nodal status in various tumors and has become the standard procedure in nodal staging of breast cancer and melanoma. However, in colorectal cancer, sentinel lymph node detection techniques are still controversial as the sensitivity reported in the literature varies from one study to another. Recently, indocyanine green fluorescence–guided surgery has been reported to be a useful technique for detection of macroscopic and microscopic metastatic deposits in lymph nodes after intravenous administration of indocyanine green dye. However, no studies have focused on the potential role of sentinel lymph node detection after systemic administration of indocyanine green dye, so-called systemic sentinel lymph nodes, or on the correspondence between the identification of the sentinel lymph node by standard local injection techniques and the detection of fluorescent lymph nodes with this new approach.

**Objective:**

The aim of this protocol is to validate the concept of sentinel lymph nodes identified by fluorescence imaging after intravenous injection of indocyanine green dye and to compare the sentinel lymph nodes identified by fluorescence imaging with sentinel lymph nodes detected by the standard blue dye technique.

**Methods:**

This study (SeLyNoFI; Sentinel Lymph Nodes Fluorescence Imaging) is a diagnostic, single-arm, open-label feasibility study, including patients with colorectal adenocarcinoma with or without metastatic disease who are admitted for elective colorectal resection of the primary tumor. This study evaluates the feasibility of a new approach for improving the accuracy of nodal staging using fluorescence imaging after intravenous administration of indocyanine green dye. Sensitivity, positive predictive value, and accuracy of the classical blue dye technique and of the investigatory fluorescence imaging technique will be calculated. Translational research will be proposed, if applicable.

**Results:**

As of June 2020, this study has been registered. Submission for ethical review is planned for September 2020.

**Conclusions:**

The potential correlation between the two different approaches to detect sentinel lymph nodes offers new strategies for improving the accuracy of nodal staging in colorectal cancer. This new concept of the systemic sentinel lymph node and a greater understanding of the interactions between systemic sentinel lymph nodes and standard sentinel lymph nodes may provide important information regarding the underlying mechanism of primary tumor lymphatic drainage. The enhanced permeability and retention effect can also play a role in the fluorescence of systemic sentinel lymph nodes, especially if these lymph nodes are inflamed. In this case, we can even imagine that this new technique will highlight more instances of lymph node–positive colorectal cancer.

**International Registered Report Identifier (IRRID):**

PRR1-10.2196/17976

## Introduction

Colorectal cancer represents a major cause of cancer-related mortality worldwide [1]. Nodal staging in colorectal cancer is of major concern as patients with stage III cancer, which is defined by nodal metastases at pathology, should receive adjuvant chemotherapy [2]. Sentinel lymph node analysis has been shown to accurately reflect nodal status in various tumors and sentinel lymph node detection techniques have become the standard of care in breast cancer and malignant melanoma where unnecessary lymphadenectomy can be avoided in patients with negative nodal status [3,4]. Sentinel lymph node detection was introduced for colorectal cancer in the early 2000s [5,6]. The aim of sentinel lymph node detection in colorectal cancer is not to identify the need for lymphadenectomy—this is already systematically performed during surgery—but to identify the first tumor draining node and focus on more advanced techniques during histopathological analysis to improve the accuracy of nodal staging. Despite some encouraging results, the technique has not been widely used. This is probably due to the fact that data reported in the literature have provided mixed results [5,7]. The poor performance of classical sentinel lymph node detection techniques using intra- or peritumoral injections in colorectal cancer could be related to two factors. The first is that, in patients with locally advanced tumors, draining lymphatic channels could be obstructed by the tumor, leading to false negative results [5]. The second is that mesenteric drainage is more heterogeneous when compared with cutaneous or subcutaneous region drainage, such as those involved in breast cancer or in melanoma, potentially leading to missed metastases [8]. For this reason, there may be discordance between the first lymph node, which anatomically drains the tumor and which is detected after peritumoral injection, defined classically as the *sentinel lymph node*, and lymph nodes invaded by cancer cells and detected after intravenous injection that we have defined as the *systemic sentinel lymph node*.

Recently, indocyanine green–fluorescence imaging has emerged as a potential technique for the detection of sentinel lymph nodes after peritumoral injection [9-11]. The preliminary results of studies [12-15] using peritumoral injection of indocyanine green dye for sentinel lymph node detection in colorectal cancer were encouraging, but we recently reported the results of a pilot study [16] which were disappointing in terms of accuracy and sensitivity.

New approaches are thus required. Recently, we published our observations on using fluorescence imaging after intravenous injection of indocyanine green dye to detect lymph node metastatic deposits both ex vivo and in vivo in colorectal cancer [17]. We confirmed these findings in a recent retrospective study [18] evaluating 12 patients who underwent colonic resection, for peritoneal metastasis detection after intravenous injection. Moreover, we observed that primary colonic tumors were more fluorescent than surrounding tissue upon fluorescence imaging after intravenous injection of indocyanine green dye.

**Figure 1:**
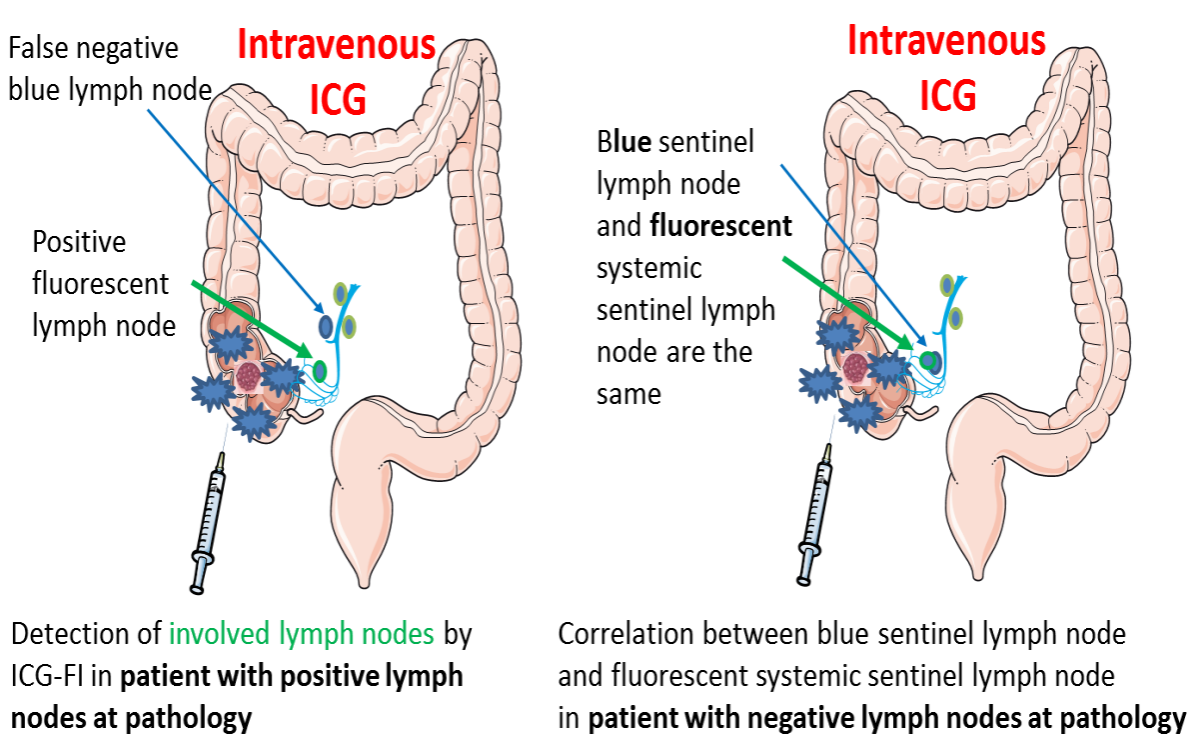
Illustrations of the systemic sentinel lymph node hypotheses. In patients with small tumors, we expect to find a correlation between the blue sentinel lymph node and the systemic lymph node. In patients with large tumors, we expected to have a false negative blue sentinel lymph node and a true positive systemic lymph node. FI: fluorescence imaging; ICG: indocyanine green.

Therefore, we hypothesized that ex vivo fluorescence imaging after intravenous injection of indocyanine green dye could represent a new approach for improving nodal staging through detection of fluorescent lymph nodes on the operative specimen. Furthermore, we hypothesized that these lymph nodes, identified after intravenous injection of indocyanine green dye and defined as systemic sentinel lymph nodes, when compared with classical anatomical tumor-draining sentinel lymph nodes, could represent a lymph node subset that is more sensitive to nodal invasion, thus serving as a more appropriate target for advanced histopathological analyses. The pathophysiological mechanism is 3-fold: first, in patients with large tumors, involved lymph nodes will be more fluorescent; second, in patients with small tumors, the fact that primary colonic tumors accumulate indocyanine green dye and that the dye is progressively cleared from the tumor by lymphatic drainage should allow the lymph node draining the tumor to be highlighted (Figure 1); and third, regarding the enhanced permeability and retention effect, we can consider that inflamed or reactive lymph nodes accumulate more indocyanine green dye and become more fluorescent [19]. This is very interesting considering that reactive lymph nodes present more risk to be invaded and are thus a key target for extensive histopathological analyses [19].

The primary objective of the SeLyNoFI study is to assess the feasibility of metastatic lymph node detection in colorectal cancer by ex vivo fluorescence imaging after intravenous administration of indocyanine green dye. In this study, we will evaluate the sensitivity, specificity, and accuracy of the technique for determining nodal status, both intraoperatively (entire fresh specimen imaging) and in the pathology department (fixed specimens).

The secondary objectives are to correlate the results of the fluorescent sentinel lymph node detection technique after intravenous injection of indocyanine green dye with sentinel lymph node detection by the standard blue dye technique, to study the link between the local lymphatic drainage pathway (classical tumor-draining sentinel lymph nodes) and the systemic pathway (systemic sentinel lymph nodes). Finally, we will evaluate the tumor-to-background fluorescence ratio of the primary colonic tumor.

## Methods

### Study Design

**Figure 2:**
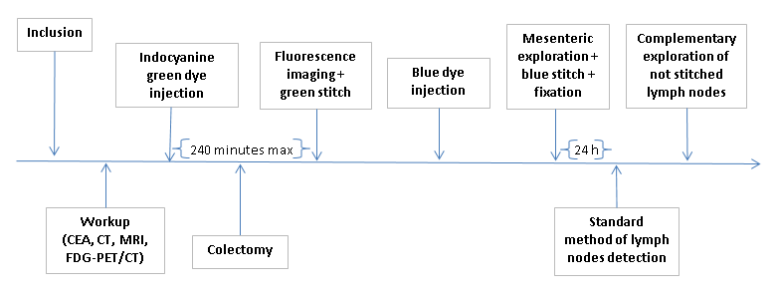
Flowchart of the study. CAE: carcinoembryonic antigen; CT: computed tomography; FDG-PET/CT: 18-Fluoro-deoxy-glucose positron emission tomography with computed tomography; MRI: magnetic resonance imaging.

The SeLyNoFI study is designed as a single-arm, 2-step (monomulticentric), academic, prospective observational-interventional study conducted by the Department of Surgical Oncology at the Institute Jules Bordet of the *Université Libre de Bruxelles* in Brussels. It will start as a monocentric, 2-stage (Simon procedure) study and will be followed by a multicentric study (among members of the International Research Institutes network) if the null hypothesis that the sensitivity of the indocyanine green–fluorescence imaging technique is lower than 60% (*p*_0_, sensitivity obtained by the classical blue dye technique improved by 10%) can be rejected based on the data generated in the monocentric study (*p*_1_, power calculated in case of a true sensitivity of 90%). To assess the feasibility of this technique, all cases will be included, even under conditions in which the technique might be expected to be unreliable (eg, long delay between injection of indocyanine green dye and fluorescence assessment). However, if the null hypothesis cannot be rejected after the monocentric phase, further development will be halted (Figure 2).

### Population

#### First Step (Monocentric)

To determine the sample size of the study, we used the 2-stage Simon procedure (minimax design) [20]. The first stage requires a small sample size (n_1_) and determines the threshold r_1_ as the number of successes (ie, TP_1_, the number of true positives) above which the trial’s second stage can begin. If that number is not surpassed (TP_1_<r_1_), then the trial ends at the end of the first step.

Once the second stage has begun, the total sample size, including those already enrolled in stage 1, is defined (n_tot_=n_1_+n_2_), and the second threshold r_tot_ for the number of successes (TP_1_+TP_2_) is defined. If the total number of successes surpasses the threshold (TP_1_+TP_2_>r_tot_), the monocentric study can terminate, and the indocyanine green dye technique will be considered worthy of further evaluation in the multicentric study. If the number of successes is not surpassed after (TP_1_+TP_2_<r_tot_ for n_tot_ cases), then the trial will terminate, and the technique will be considered inadequate and abandoned.

With *p*_0_= 60% and a *p*_1_=90%, the sample size needed in the first stage is n_1_=8 (lymph node–positive N1 or N2) and the number of successes (true positive) to surpass is r_1_=5. The total sample size needed is n_tot_=17 (lymph node–positive patients) with a total number of successes r_tot_=14 (with α=.05 and β=.10). As the prevalence of patients with N1, N2, or N3 is estimated to be 50%, the number of patients needed for enrollment in stage 1 and 2 are about 16 and 34, respectively.

Based on the number of patients treated at the Institute Jules Bordet, the time necessary to execute this step is estimated to be 1 year.

#### Second Step (Multicentric)

**Figure 3:**
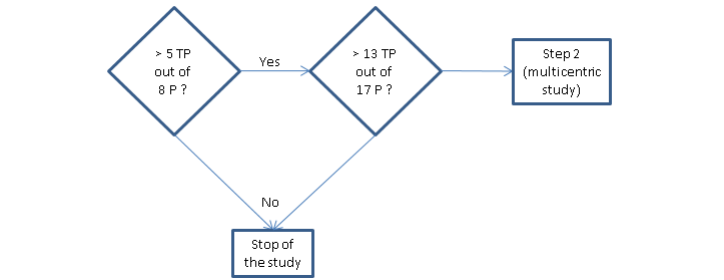
Decision diagram for 2-step study plan. True-positive patients required in stage 1 and stage 2 of the monocentric study to proceed to the multicentric study (step 2). P: patients; TP: true positive.

As the primary aim of this study is not to prove a hypothesis but to estimate parameters (sensitivity and ratio of fluorescent to nonfluorescent lymph nodes), sample size planning may be based upon the expected width of the confidence intervals [21]. In this study, in order to estimate a sensitivity of 95% with a 5% precision, we need a total sample size of 73 (lymph node–positive patients) to get a 95% confidence interval with a half-length of 5%. If the true sensitivity is 90% (ie, *p*_1_ for our Simon design), the precision will be 7% rather than 5%. As the prevalence of patients with N1, N2, or N3 is estimated to be 50%, the total number of patients needed is about 146. At least two other centers of the *Pole Hospitalier Universitaire de Bruxelles* network will be included (Figure 3).

**Figure 4:**
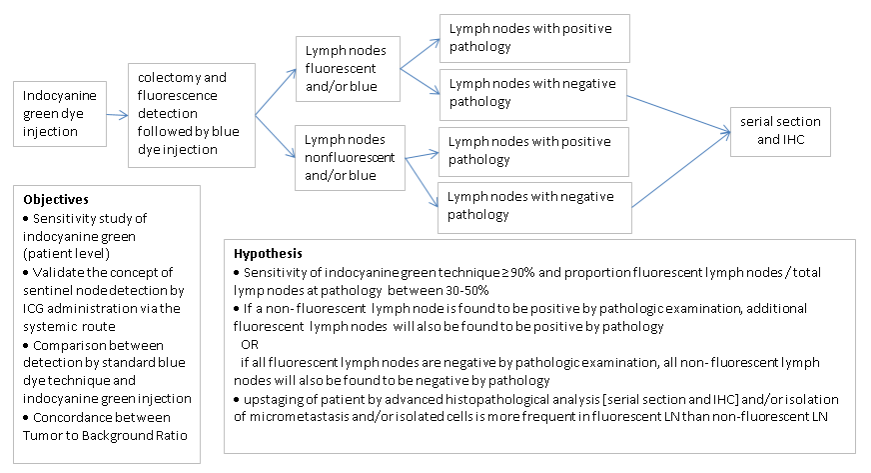
Flow of samples through the study. After indocyanine green and blue dye injection, all lymph nodes removed during colectomy will be analysed for indocyanine green fluorescence and blue dye positivity, followed by pathologic analysis. Lymph nodes that are negative by initial pathology will be further examined by serial sectioning. ICG: indocyanine green; IHC: immunohistochemistry; LN: lymph node.

The general overview of the study is shown in Figure 4.

### Inclusion Criteria

Patients with biopsy-proven primary or metastatic colorectal cancer admitted for elective surgery of the primary colorectal tumor and who provide written informed consent will be included.

### Exclusion Criteria

Patients who are younger than 18 years old; are unable to give informed consent; have a history of allergy or hypersensitivity to investigational product (active substance or ingredients), to iodine, or to shellfish; have apparent hyperthyroidism, autonomous thyroid adenoma, unifocal, multifocal, or disseminated autonomy of the thyroid gland; have documented coronary disease or advanced renal insufficiency (creatinine >1.5 mg/dL); are on concurrent medication which reduces or increases the elimination of indocyanine green dye (ie, anticonvulsants, haloperidol, and heparin) during the 2 weeks before the expected operation; are pregnant; or are breastfeeding will be excluded.

### Preoperative Work-up and Surgery

All patients will undergo standard work-up including laboratory testing for tumoral carcinoembryonic antigen, thoraco-abdominopelvic computed tomography (CT), and abdominal magnetic resonance imaging (MRI), as necessary (eg, in patients with renal insufficiency). Patients with suspected metastases will undergo 18F-fluorodeoxyglucose positron emission tomography with computed tomography (FDG-PET/CT). Patients will undergo laparoscopy or laparotomy following the standard procedures for colectomy.

### Tracer Preparation

Indocyanine green dye (Pulsion Medical Systems SE) will be diluted with 10 mL of sterile water (2 mg/mL), and a dose of 0.25 mg/kg will be administrated by slow intravenous injection by central venous catheter at the beginning of the surgical procedure.

### In the Operating Room

#### Fluorescence Imaging

After colonic and rectal resection (as necessary), the operative specimen will be placed on a back table and the mesentery exposed. Fluorescence imaging is carried out with a dedicated near-infrared camera system. A light-emitting diode light source set to a wavelength of 760 nm is used, and the detector is a charge-coupled device (CCD) camera with a filter set to detect light with a wavelength of <820 nm. The fluorescent signal is sent to a digital video processor to be displayed on a monitor in real time. Videos will be recorded on a personal computer using a standard high-definition video program. The camera will be held directly by the surgeon at a distance of approximately 20 cm from the operative specimen. During fluorescence imaging exploration, hyperfluorescent lymph nodes will be marked with a green stitch. Thereafter, the colon will be opened, and primary tumors will be imaged for their fluorescence.

#### Time From Injection to Fluorescence Imaging

The time from injection of indocyanine green dye to fluorescence imaging will be recorded. We predict that there will be a minimum of two groups of patients for analysis of the results of fluorescence imaging in view of the variability of operation durations associated with the procedure (eg, <180 minutes versus >180 minutes). Patients with prolonged operative procedures and complete clearance of indocyanine green dye from the operative specimen will be excluded from further analysis. The limit of the timing for fluorescence imaging after indocyanine green injection has yet to be determined.

#### Standard Sentinel Lymph Node Detection (Blue Dye Technique)

After fluorescence imaging in the operating room, a submucosal injection of 0.5 mL of patent blue dye will be injected at the 4 cardinal points around the primary tumor, and the injected area will be gently massaged to increase diffusion of the blue dye. After a few minutes, the mesentery will be explored for blue sentinel lymph nodes. Sentinel lymph nodes will be marked with a blue stitch.

### In the Pathology Department

#### Fluorescence Imaging

The operative specimen will be fixed and will be examined 24 hours later using standard methods for lymph node detection. Lymph nodes marked with a blue (blue sentinel lymph node) or green (fluorescent lymph node) stitch in the operating room will be examined separately and noted as blue or fluorescent lymph nodes. Concomitant blue and fluorescent lymph nodes will be noted as concordant sentinel lymph nodes for the two techniques.

Thereafter, nonstitch sentinel lymph nodes will be placed into cassettes and systematically examined for their blue or fluorescent staining and noted as fluorescent, blue, or neither blue nor fluorescent. Importantly, those stained lymph nodes found to be blue or fluorescent afterwards will not be classified as sentinel lymph nodes.

Finally, the operative specimen will be examined with the camera for complementary exploration and to evaluate whether fluorescence imaging is able to detect more lymph nodes than classical analyses can detect. Those lymph nodes will be categorized as *clinically unfound lymph nodes*.

#### Pathology Examination

All lymph nodes will be placed into cassettes. Nonsentinel lymph nodes (including blue stained lymph nodes found afterwards) will undergo classic pathologic analysis (longitudinal bivalve section). If lymph nodes have a size of less than 4 mm, they will be loaded unsectioned into the cassette. Conversely, blue sentinel lymph nodes and all fluorescent lymph nodes (found to be fluorescent in the operating room and in the pathology department) will be sectioned into multiple slices at 2 mm to 3 mm intervals along their longest axis. Thereafter, all cassettes will be paraffin embedded. All lymph nodes will be analyzed after standard staining with hematoxylin and eosin.

Negative sentinel lymph nodes and fluorescent lymph nodes will be examined with further serial sectioning at 150 µm intervals and evaluated by standard hematoxylin and eosin staining.

To avoid the risk of bias with micrometastases being detected more frequently in fluorescent lymph nodes related to the higher number of examined fluorescent lymph nodes in comparison with nonfluorescent lymph nodes, a similar number of negative lymph nodes (nonstained or blue found afterward at pathology) will be examined in the same way as the fluorescent lymph nodes by serial section with standard hematoxylin and eosin staining.

### Semiquantitative Image Analysis of Fluorescence

Recorded videos will be used to calculate the fluorescence intensity of all primary tumors and analyzed lymph nodes. Regions of interest will be drawn over the primary tumor and lymph nodes and over the adjacent background tissue. Fluorescence intensity, expressed in arbitrary units of lymph nodes and tumor background, will be measured with the IC-Calc (version 2.0, Pulsion Medical Systems SE). Finally, tumor-to-background fluorescence ratios will be calculated for each lymph node and for the primary tumor. Fluorescence imaging videos of fresh (nonfixed) and fixed lymph nodes in cassettes will be used for tumor-to-background fluorescence ratio calculations. For primary colon calculations, tumor-to-background fluorescence ratio will be calculated using intraoperative videos on the entire fresh operative specimen after exposure of the colon.

All specimens will be imaged under standard conditions with the near-infrared camera by the same person. The findings of these images will be correlated with definitive pathological reports of the lymph nodes.

### Statistical Analyses

The sensitivity of the first step (monocentric study) will be evaluated for patients where the fluorescence evaluation has been performed just after the colectomy in the operating room and also afterward in the pathology department.

Sensitivity and positive predictive value will be computed at the patient level, on the total, and in different subgroups based on histology of the tumor (nonmucinous versus mucinous adenocarcinoma) or delay between injection of indocyanine green dye and fluorescence examination.

Upgrading percentage will be calculated on fluorescent lymph nodes with negative pathological examination. Concordance between visual scale and tumor-to-background fluorescence ratio will be evaluated with the kappa statistic.

### Ethical Considerations

The study will be submitted by the principal investigator, the national coordinator, or the sponsor (or its legal representative), in accordance with local regulations, to and approved by an appropriate independent ethical review committee or institutional review board and a regulatory authority if required by the national laws of the countries where the study will be conducted. Local regulatory approval may also be required.

The study will not start at a participating site before written approval by the corresponding ethics committee has been obtained and the local regulatory requirements have been complied with.

The principal investigator and the sponsor will ensure that the study is conducted in full conformance with the principles of the Declaration of Helsinki 1964, as revised from time to time and with the laws and regulations of the country in which the research is conducted, whichever affords the greater protection to the individual. The study must fully adhere to the principles outlined in Guideline for Good Clinical Practice ICH-E6 Tripartite Guideline and with national laws.

For studies conducted in European Union or European Economic Area countries, the principal investigator will ensure compliance with the EU Clinical Trial Directive (2001/20/EC) and with the EU Data Protection Directive (95/46/EC).

In other countries where guidelines for good clinical practice exist, the sponsor and the principal investigators will strictly ensure adherence to the stated provisions.

## Results

The study was registered in the European Union Drug Regulating Authorities Clinical Trials Database (Eudract number 2020-002521-29) in June 2020. Submission for ethical review is planned for September 2020.

## Discussion

Accurate nodal staging is crucial in colorectal cancer as a prognostic factor and to determine the need for adjuvant treatment. In patients with nodal invasion (stage III), adjuvant chemotherapy is required, while the benefit of adjuvant chemotherapy in stage II remains controversial [2]. Notably, 20% of patients identified as stage II colorectal cancer will experience recurrence, potentially due to missed lymph node metastases [2], justifying efforts to increase the accuracy of nodal staging. It has been clearly demonstrated that one of the most important prognostic factors associated with the accuracy of nodal staging is the number of lymph nodes analyzed from the operative specimen [22], and the Union for International Cancer Control recommends that at least 12 lymph nodes should be resected and analyzed [23]. In the early 2000s, sentinel lymph node detection in colorectal cancer emerged as a promising technique for increasing the accuracy of nodal staging, focusing pathological analyses on detection of micrometastases in a limited number of lymph nodes using advanced techniques such as serial section, immunochemistry, and reverse transcription polymerase chain reaction techniques [6]. Currently, however, the contribution of classical sentinel lymph node detection with blue dye in colorectal cancer remains a subject of debate [5,7,8]. One of the major limitations of this technique that uses intra- or peritumoral marker injection is that it may result in false negatives due to the fact that lymphatic drainage can be impaired by tumor compression in large tumors (pT3 and pT4) [5]. Therefore, the technique is mostly used for staging smaller tumors. This is inconsistent with the higher risk for nodal dissemination associated with large tumors compared to small tumors. Recently, the use of fluorescence imaging in the detection of sentinel lymph nodes has emerged as a promising technique in several cancers [9,11-16], but the sensitivity of the blue dye method in patients with stage pT3 or pT4 tumors remains disappointing [16]. This provides the rationale for finding a solution that overcomes this problem, such as using intravenous injection as we recently reported in a proof-of-concept study in metastatic colorectal cancer [17,18].

The purpose of this study is to evaluate a new technical approach using fluorescence imaging after systemic administration of indocyanine green dye in order to increase nodal staging accuracy in colorectal cancer. Our working hypotheses are that fluorescence imaging may be able to detect metastatic lymph nodes and that hyperfluorescent lymph nodes detected after intravenous administration of the dye (systemic sentinel lymph nodes) will be more representative of cancer invasion than the classical sentinel lymph nodes detected after local peritumoral injection. In that sense, we will correlate the lymph nodes identified using fluorescence techniques with lymph nodes found by the classical blue dye sentinel lymph node detection technique.

We expect that more fluorescent lymph nodes will be found than sentinel lymph nodes, but based on current experience in breast cancer, this number is still largely inferior to the total number of lymph nodes resected and analyzed on the operative specimen, allowing advanced histopathological analyses on only a limited number of lymph nodes. In this study, we propose to include both metastatic and nonmetastatic colorectal patients as the principal objective is to demonstrate the feasibility of the concept.

This observational study was designed to evaluate the feasibility of a new concept that aims to increase the accuracy of nodal staging using fluorescence imaging after intravenous administration of indocyanine green dye in colorectal cancer patients. Furthermore, this study will serve to evaluate the validity of the concept of the systemic sentinel lymph node compared to the classical anatomical sentinel lymph node, draining directly from the primary tumor site, in the context of colorectal cancer.

## Abbreviations

CT: computed tomography
FDG-PET/CT: 18-Fluoro-deoxy-glucose positron emission tomography with computed tomography
MRI: magnetic resonance imaging
PET: positron emission tomography

## References

[1] (2015). United States cancer statistics incidence and mortality web-based report. United States Cancer Statistics CDC.

[2] André T, de Gramont A, Vernerey D, Chibaudel B, Bonnetain F, Tijeras-Raballand A, Scriva A, Hickish T, Tabernero J, Van Laethem JL, Banzi M, Maartense E, Shmueli E, Carlsson GU, Scheithauer W, Papamichael D, Möehler M, Landolfi S, Demetter P, Colote S, Tournigand C, Louvet C, Duval A, Fléjou J, de Gramont A (2015). Adjuvant Fluorouracil, Leucovorin, and Oxaliplatin in Stage II to III Colon Cancer: Updated 10-Year Survival and Outcomes According to BRAF Mutation and Mismatch Repair Status of the MOSAIC Study. J Clin Oncol.

[3] Dummer R, Hauschild A, Guggenheim M, Jost L, Pentheroudakis G, ESMO Guidelines Working Group (2010). Melanoma: ESMO Clinical Practice Guidelines for diagnosis, treatment and follow-up. Ann Oncol.

[4] Lyman GH, Temin S, Edge SB, Newman LA, Turner RR, Weaver DL, Benson AB, Bosserman LD, Burstein HJ, Cody H, Hayman J, Perkins CL, Podoloff DA, Giuliano AE, American Society of Clinical Oncology Clinical Practice (2014). Sentinel lymph node biopsy for patients with early-stage breast cancer: American Society of Clinical Oncology clinical practice guideline update. J Clin Oncol.

[5] Liberale G, Lasser P, Sabourin J, Malka D, Duvillard P, Elias D, Boige V, Goéré D, Ducreux M, Pocard M (2007). Sentinel lymph nodes of colorectal carcinoma: reappraisal of 123 cases. Gastroenterol Clin Biol.

[6] Saha S, Bilchik A, Wiese D, Espinosa M, Badin J, Ganatra BK, Desai D, Kaushal S, Singh T, Arora M (2001). Ultrastaging of colorectal cancer by sentinel lymph node mapping technique--a multicenter trial. Ann Surg Oncol.

[7] van der Pas MH, Meijer S, Hoekstra OS, Riphagen II, de Vet HCW, Knol DL, van Grieken NCT, Meijerink WJHJ (2011). Sentinel-lymph-node procedure in colon and rectal cancer: a systematic review and meta-analysis. Lancet Oncol.

[8] Shiozawa M, Akaike M, Yamada R, Godai T, Yamamoto N, Saito H, Sugimasa Y, Takemiya S, Rino Y, Imada T (2007). Clinicopathological features of skip metastasis in colorectal cancer. Hepatogastroenterology.

[9] Hirche C, Murawa D, Mohr Z, Kneif S, Hünerbein M (2010). ICG fluorescence-guided sentinel node biopsy for axillary nodal staging in breast cancer. Breast Cancer Res Treat.

[10] Gilmore DM, Khullar OV, Gioux S, Stockdale A, Frangioni JV, Colson YL, Russell SE (2013). Effective low-dose escalation of indocyanine green for near-infrared fluorescent sentinel lymph node mapping in melanoma. Ann Surg Oncol.

[11] Rossi EC, Ivanova A, Boggess JF (2012). Robotically assisted fluorescence-guided lymph node mapping with ICG for gynecologic malignancies: a feasibility study. Gynecol Oncol.

[12] Kusano M, Tajima Y, Yamazaki K, Kato M, Watanabe M, Miwa M (2008). Sentinel node mapping guided by indocyanine green fluorescence imaging: a new method for sentinel node navigation surgery in gastrointestinal cancer. Dig Surg.

[13] Hirche C, Mohr Z, Kneif S, Doniga S, Murawa D, Strik M, Hünerbein M (2012). Ultrastaging of colon cancer by sentinel node biopsy using fluorescence navigation with indocyanine green. Int J Colorectal Dis.

[14] Noura S, Ohue M, Seki Y, Tanaka K, Motoori M, Kishi K, Miyashiro I, Ohigashi H, Yano M, Ishikawa O, Miyamoto Y (2010). Feasibility of a lateral region sentinel node biopsy of lower rectal cancer guided by indocyanine green using a near-infrared camera system. Ann Surg Oncol.

[15] Nagata K, Endo S, Hidaka E, Tanaka J, Kudo S, Shiokawa A (2006). Laparoscopic sentinel node mapping for colorectal cancer using infrared ray laparoscopy. Anticancer Res.

[16] Liberale G, Vankerckhove S, Galdon MG, Larsimont D, Ahmed B, Bouazza F, Moreau M, Nakadi IE, Donckier V, Bourgeois P (2016). Sentinel Lymph Node Detection by Blue Dye Versus Indocyanine Green Fluorescence Imaging in Colon Cancer. AR.

[17] Liberale G, Vankerckhove S, Galdon MG, Donckier V, Larsimont D, Bourgeois P (2015). Fluorescence imaging after intraoperative intravenous injection of indocyanine green for detection of lymph node metastases in colorectal cancer. Eur J Surg Oncol.

[18] Liberale G, Galdon MG, Moreau M, Vankerckhove S, El Nakadi I, Larsimont D, Donckier V, Bourgeois P (2016). Ex vivo detection of tumoral lymph nodes of colorectal origin with fluorescence imaging after intraoperative intravenous injection of indocyanine green. J Surg Oncol.

[19] Maeda H, Wu J, Sawa T, Matsumura Y, Hori K (2000). Tumor vascular permeability and the EPR effect in macromolecular therapeutics: a review. Journal of Controlled Release.

[20] Simon R (1989). Optimal two-stage designs for phase II clinical trials. Control Clin Trials.

[21] Röhrig B, du PJ, Wachtlin D, Kwiecien R, Blettner M (2010). Sample Size Calculation in Clinical Trials (Part 13 of a Series on Evaluation of Scientific Publications). Dtsch Arztebl Int 2010; 107(31–32): 552–6.

[22] Hida J, Yasutomi M, Maruyama T, Fujimoto K, Uchida T, Okuno K (1997). The extent of lymph node dissection for colon carcinoma: the potential impact on laparoscopic surgery. Cancer.

[23] Sobin L, Gospodarowicz M, Wittekind C (2009). TNM Classification of Malignant Tumours, 7th Edition. Wiley-Blackwell.

